# Seismic performance analysis of braced steel structures based on vibration experiments and finite element simulation

**DOI:** 10.1371/journal.pone.0322379

**Published:** 2025-05-09

**Authors:** Nan Cui, Shuangshuang Liu

**Affiliations:** 1 Department of Civil Engineering, Changzhi Vocational and Technical College, Changzhi, China; 2 School of Civil Engineering and Architecture, Baise University, Baise, China; National University of Sciences and Technology, PAKISTAN

## Abstract

The study analyzes the seismic performance of spatial steel frame structures by creating a three-dimensional nonlinear finite element model and designing vibration table model experiments. This is done to ensure the seismic performance of the steel framing-bracing system in spatial steel frame structures and to raise the level of seismic and disaster prevention technology of steel structures. The outcomes revealed that under the experimental load consisting of simulated seismic waves generated by the shaker, the initial natural frequencies of the bidirectional bracing arrangement in white noise in the X and Y-directions were larger, 53.18 Hz and 72.49 Hz, respectively. The decrease of the vibration frequency of the bidirectional bracing arrangement structure was smaller under the increase of the excitation acceleration. In El Centro seismic waves, Taft seismic waves and Wenchuan seismic waves, the changes of damping ratio, acceleration amplification factor and relative displacement of bidirectional bracing arrangement were smaller than those of unidirectional bracing arrangement. The bidirectional bracing was beneficial to the overall stiffness and seismic performance of the structure, and helps the steel frame structure to resist lateral displacement. At the same time, the bidirectional support was more favorable to the seismic performance if the support structure was arranged according to the wave, which could effectively reduce the strain. Under the simulated seismic action of the 3D non-linear finite element model, the finite element model results were tested by inputting the parameters of Wenchuan seismic wave, the fit between the finite element model results and the vibration table experiment results was high, and the acceleration time-course curve and displacement time-course curve were basically the same. The simulation errors of the maximum values of acceleration and displacement were 7.0% and 4.0%, and some of the larger errors were within the acceptable error range. This study provides reference and theoretical guidance for the research on the seismic performance of steel framing - bracing system under seismic action, which is conducive to the expansion of the application field of steel seismic structures.

## Introduction

Earthquake disasters have become more commonplace worldwide in recent times. The safety of people’s lives and property is significantly threatened by the suddenness and strong destructiveness of earthquakes. Along with the scale and complexity of urban buildings, the possibility of high-rise structure collapse and damage caused by earthquakes is gradually increasing, and it is necessary to improve the level of earthquake mitigation of building structures [1,2]. At present, steel structure (SS) has gradually become the main form of building structure due to its features of good stiffness, high strength, light weight and easy construction. At the same time, compared with the traditional concrete structure, SS buildings have higher tensile, compressive and flexural strength. The structure can withstand large loads, has good seismic performance (SP), and has significant advantages in seismic design. Steel framing-bracing (SFB) system is a widely used structural system for multiple and high-rise steel buildings with high strength and good ductility. The arrangement of SFBs within the SS can augment the structure’s overall stiffness and load carrying capability [3,4].

Two lines of defense against external loads are the bracing and frame, and it is crucial to carefully plan the type and arrangement of the bracing [5,6]. However, in actual SS projects, the design of bracing arrangement involves complex structural mechanics and engineering construction experience, and needs to consider the overall stability, stiffness, bearing capacity and other factors at the same time. The traditional arrangement design method relying on industry experience and structural trial calculations is difficult to achieve the optimal solution of the bracing arrangement design [7,8]. Moreover, the existing steel frame bracing arrangement design is mostly studied in the form of plane frame. However, purely plane frame research will weaken the designer’s judgment on the coupling effect and SP of the structural frame under seismic action. In order to fill the gap in the design of bracing arrangement for spatial structures, the seismic analysis of different forms of braced spatial steel frame (SSF) is carried out by combining scale model (SM) experiments and numerical 3D model experiments.

The study fills the gap of SP research on SSF structures by combining vibration table test (VTT) and finite element (FE), which enriches the level of theoretical research on SFB system in the field of seismic mitigation technology. The study is divided into four parts in total. The first part accomplished a review of the current state of research related to structural seismic and SS bracing systems. In the second part, the VTT of steel frame was designed and the numerical model of steel frame structure was constructed. The third part analyzed the shaking table and FE experiments. Part IV summarizes the main conclusions and future work of the study. The study is expected to provide scientific and reasonable guidance for SP upgrading of multiple and high-rise SS and to advance the application of SFB system in seismic resistance.

## 1. Related works

With the advancement of urbanization development process, the application of multiple and high-rise SS has been developed rapidly, and the enhancement of its SP has received extensive attention from scholars. Bae J et al. explored the elastic enhancement effect of visco-plastic braced dampers on SS buildings. Numerical simulation outcomes revealed that the viscoplastic brace can effectively control the drift and plastic deformation of structural members and exhibit strong SP in SS buildings. Meanwhile, the viscoplastic brace can control the peak force of the structure, reduce the peak ground acceleration and velocity, and effectively reduce the structural damage [9]. Concentric braced frame (CBF) lateral system has been widely used in SS. However, the stability of CBF is poor in the face of higher magnitude earthquakes. Jalalvandi M et al. introduced shape memory alloy (SMA) and buckling restrained braces (BRB) to improve the SP of braced steel frames in CBF. The results of nonlinear time course analysis by OpenSEES showed that both SMA and BRB bracing provide energy dissipation and reduce the maximum interstory drift. SMA also reduced the permanent displacement of the structure [10]. While BRB works well for the SP of moment resisting frames (MRFs), there is still a shortage of advice in the current methodologies to increase performance. Gutiérrez-Urzúa F et al. evaluated the SP of different retrofitting options of BRB through incremental dynamic analysis to improve the overall performance of the structure [11].

Paronesso M et al. connected a steel CBF system to the diaphragm of a gravity frame system in order to investigate the function of sliding friction dampers (SFDs) as dissipative floor connections for SP improvement in steel CBF structures. The outcomes of nonlinear response history analysis showed that the defined activation force of SFDs effectively mitigated the high modal effects, prevented the concentration of interstory drift, and reduced the absolute acceleration demand [12]. Industrial facilities’ seismic hazard assessments have primarily depended on historical data and dissociated secondary member analysis, ignoring the dynamic interplay between the main structure and process machinery. Nardin C et al. explored the interaction between an extended multistory braced frame SS and nonstructural members. A site-based synthetic ground vibration model was used to excite the vibration cycles of nonstructural members and to analyze the SP of nonstructural members of a multilayered steel MRFs for the blast furnace industry. The experimental findings demonstrated that the structure was bending and that there was significant interaction between the blast furnace’s bottom plate beams and vertical storage tanks [13]. Modular building is a new type of environmentally friendly building technology for on-site assembly. However, the typical lateral load-bearing system codes are primarily referenced in the seismic and detailed design of angle-supported modular structures. Regarding this, using nonlinear simulation approaches, Farajian M et al. performed an analysis of the SP affecting factors of modular corner-supported steel bracing systems that are four, eight, and twelve stories tall. The experimental results indicated that dynamic loading induced inelastic force redistribution. It was suggested that the response modification factor for supporting the corner braced modular system should be taken as 2.5, which is higher than that taken by conventional structural codes [14].

Hu S et al. created a novel kind of self-centering SMA bracing to increase the SP and self-centering capability of steel frame constructions. According to the testing findings, the steel frame with slip support had a lower interstory drift ratio, roof displacement, and roof acceleration than the naked steel frame with slip support. The self-centering SMA support achieved a better hysteresis curve in the steel frame with good energy dissipation and centering ability [15]. One innovative method to improve a structure’s SP is to create plastic hinges inside its flexible sections. Mahdavi M et al. established ten different SS bracing systems based on Sap2000 software, containing two types of steel frames, 4-story and 8-story. The experimental results showed that concentrically braced 4-storey and zipper braced 8-storey will produce the maximum plastic hinge capacity [16]. Mahdavi M et al. built 5-, 8- and 11-layer SSs with irregular mass and height based on SAP2000 software, and the SSs contain three different types of concentric bracing. 11 near-fault and far-fault scaled seismic analyses were performed using direct integration method. The knee bracing outperformed the inverted V bracing in terms of shear characteristics and the knee bracing in terms of the structure’s acceleration and displacement parameters, according to the experimental data [17]. In view of the fact that limited beam yielding can increase the interstorey drift capacity of a shaped braced frame fracture, Orgev A A et al. proposed a new zigzag frame design for yielding beam shaped braced frames and performed SP analysis in a 9-storey structure. The experimental results showed that the zigzag frame design has a large interstory drift ratio and is economical [18].

In summary, domestic and international research on SS bracing system and seismic technology mainly centers on seismic measures and bracing forms, and the research on SP under seismic action has made good progress. However, the seismic resistance of SS under earthquakes is mostly in the form of plane frames, while actual projects are presented in the form of space structures. In this study, the bracing arrangement design of SSF is investigated.

## 2. Seismic performance study of spatial steel frames

The study takes a steel frame structure in space form as an example, and analyzes the SP of different bracing arrangement forms under seismic action through VTT analysis and 3D numerical modeling.

### 2.1. Scaled model design of spatial steel frame

**Fig 1:**
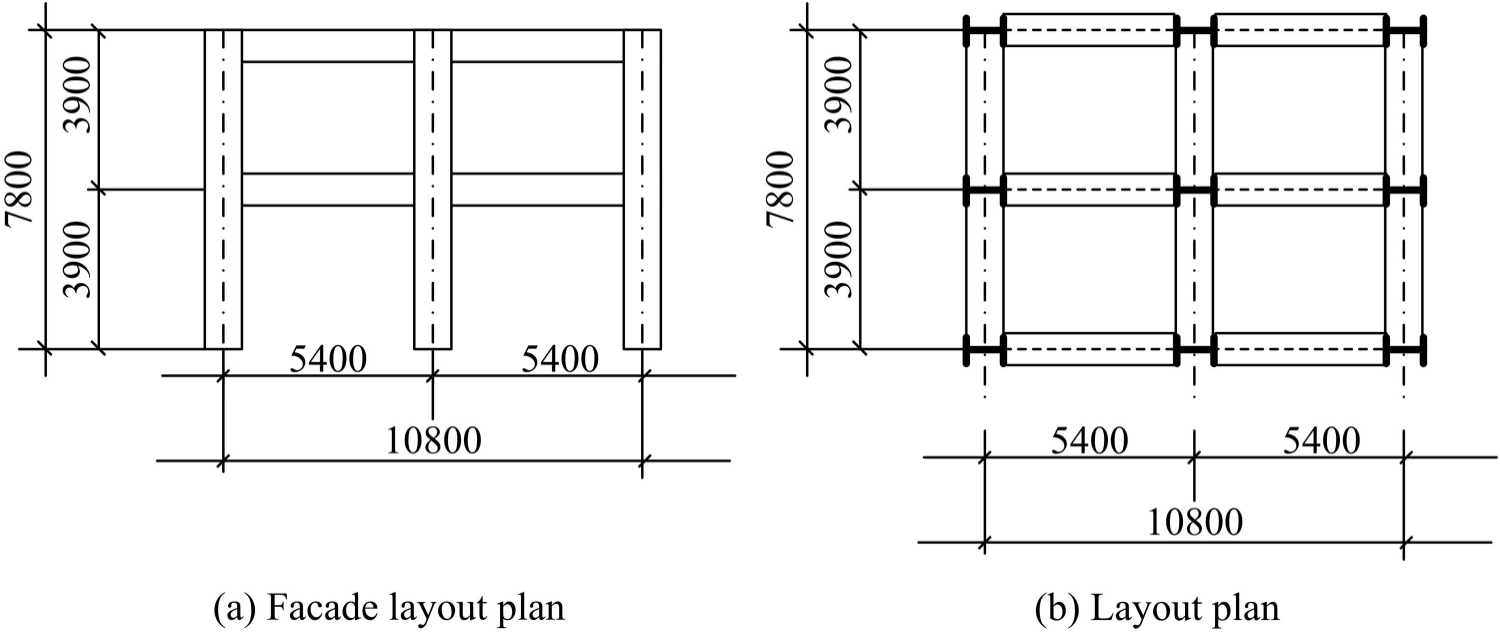
Spatial Dimension Relationship Diagram of the Prototype Structure of the Spatial Steel Frame.

The study chooses SM experiments corroborated with finite element simulation (FES) to carry out the SP analysis, starting with the design of the SM experiments. The facade and plan structure configuration of the SSF prototype structure used in the study is shown in Fig 1.

In Fig 1, the prototype structure is a two-story, two-span, two-bay structure with a span of 5.4 m and a storey height of 3.90 m. The design seismic intensity (SI) of the structure is 8 degrees. The design fundamental seismic acceleration value is 0.20 g, and the slope angle of the area where the structure is situated is 8 degrees. According to the *Code for Seismic Design of Buildings*, the design seismic grouping is determined to be the first group. The structure is subjected to roof constant load and floor constant load of 5.5kN/m^2^. The roof live load is 0.5kN/m^2^ and the floor live load is 3.0kN/m^2^. The thickness of the floor slab is 120mm. The structure is constructed by using reinforced concrete with concrete grade C40 and steel reinforcement HRB400. The beams and columns of the entire two-story, two-span, two-bay SSF are Q235B steel, and the hot-rolled H-beam HN1000*300*19*36mm is used. The cross-sectional area is 395.10, and the tensile strength is between 370MPa-480MPa. The yield strength is 284MPa, and the quality grade is A grade. Among them, the support member adopts HPB235 bare round mild steel, Ф200 × 8.

It is examined that the scaled-down design is carried out with the aid of the principles of stiffness equivalency and similarity in order to simplify the analysis and design of the prototype structure. The similarity principle refers to the laws and theorems that the physical phenomena between the prototype and the model have certain similarities. When the model and prototype follow the similarity theorem, the corresponding values of the prototype can be deduced from the experimental results of the model [19,20]. The research generates self-similar SMs, and the prototype structure’s stresses, strains, and structural damage forms are lessened when SMs are seismically loaded. The second theorem of similarity shows that the solutions of all similar physical phenomena must be described by the same characteristic number of relational equations, which are used to establish physical equations using physical quantities and further derived to obtain the dimensionless relational equations [21,22]. Therefore, it is investigated to calculate the relational equations for the different parameters of the model using measure analysis to obtain the similar proportionality relationship between the prototype and the model. In this process, the study assumes that the materials used in the prototype and the model are similar in physical properties, i.e., the ratio of key material parameters such as modulus of elasticity, Poisson’s ratio, and density remain constant between the prototype and the model. As the strength of the material is size-dependent, however, the study assumes that the strength properties remain consistent during scaling and that the microstructure of the material has a negligible effect on the behaviour of the model. However, during the actual experimental operation, the size effect and the limitation of manufacturing accuracy of the material properties can easily lead to the inherent limitations of the model design, which need to be treated with caution during the unfolding of the experiments.

The model length similarity ratio $S_{l}$ is determined by the prototype structure with SM experimental conditions. The modulus of elasticity similarity ratio $S_{E}$ is determined by the steel material used in the SSF. The research setup $S_{E}=1$. Moreover, the model design process assumes that the vertical compressive strain of the model and the prototype are the same, i.e., the vertical compressive stress ratio $S_{\sigma }$ usually satisfies $S_{\sigma }=S_{E}$. The calculation of the mass similarity ratio $S_{m}$ satisfies Equation (1).


$$S_{m}=S_{E}S_{l}^{2}=S_{\sigma }S_{l}^{2}$$ (1)

However, to ensure the reduction of the SM to the seismic response and to try to ensure that the vertical compressive stresses are consistent with those of the prototype structure, the study adopted the under-artificial mass model weighting method [23]. Therefore, the calculation of the stress similarity ratio $S_{\sigma }$ needs to be inversely calculated from the mass similarity ratio $S_{m}$. The overall mass of the prototype structure and the modeled structure’s mass after undermass counterweighting are used to calculate the mass similarity ratio $S_{m}$. Then $S_{\sigma }$ is calculated by backpropagation according to the relationship Equation (1).

The study continues to derive other similar relationships for material properties, geometric properties, loading, and dynamic properties based on $S_{l}$, $S_{E}$, and $S_{\sigma }$. In addition, in order to correct for the SP discrepancy brought about by the under-artificial mass model counterweighting method, the study introduces the stiffness ratio $S_{K}$ and the frequency ratio $S_{f}$ to correct for the peak acceleration and vibration hold-up of seismic waves (SW) in the modeling experiments [24]. The calculation procedure of $S_{K}$ and $S_{f}$ is shown in Equation (2).


$$\left\{ \begin{array}{c} S_{K}=S_{E}S_{l}\\ S_{f}={\left[S_{E}/\left(S_{\sigma }S_{l}\right)\right]}^{\frac{1}{2}} \end{array}\right.$$ (2)

The principle of stiffness equivalence means that the stiffness of the prototype can be replaced by an equivalent simplified model, and the section size (SecS) of the calculated structural member can be derived from the principle of stiffness equivalence [25]. The flexural stiffness $E^{p}I^{p}$ and tensile and compressive stiffness $E^{p}A^{p}$ of the prototype satisfy the relationship Equation (3).


$$\left\{ \begin{array}{c} \frac{E^{m}I^{m}}{E^{p}I^{p}}=\frac{E_{D}^{m}I_{D}^{m}}{E^{p}I^{p}}=S_{E}S_{l}^{4}\\ \frac{E^{m}A^{m}}{E^{p}A^{p}}=\frac{E_{D}^{m}A_{D}^{m}}{E^{p}A^{p}}=S_{E}S_{l}^{2} \end{array}\right.$$ (3)

In Equation (3), $E_{D}^{m}I_{D}^{m}$ and $E_{D}^{m}A_{D}^{m}$ denote the flexural, tensile and compressive stiffnesses designed according to the similarity principle, respectively. $E^{m}I^{m}$ and $E^{m}I^{m}$ represent the actual flexural, tensile and compressive stiffnesses, respectively. Since the model is consistent with the prototype material, $E^{m}$ and $E^{p}$ are equal. According to Equation (3), the SecS calculation of the member is derived as shown in Equation (4).


$$\left\{ \begin{array}{c} \frac{I^{m}}{I^{p}}=S_{l}^{4}\\ \frac{A^{m}}{A^{p}}=S_{l}^{2} \end{array}\right.$$ (4)

The SecS of slant support of the structure can be found from Equation (4) and the calculation procedure is shown in Equation (5).


$$\left\{ \begin{array}{c} \frac{{\left(D^{m}\right)}^{4}\left[1-{\left(a^{m}\right)}^{4}\right]}{{\left(D^{p}\right)}^{4}\left[1-{\left(a^{p}\right)}^{4}\right]}=S_{E}S_{l}^{4}\\ \frac{{\left(D^{m}\right)}^{2}\left[1-{\left(a^{m}\right)}^{2}\right]}{{\left(D^{p}\right)}^{2}\left[1-{\left(a^{p}\right)}^{2}\right]}=S_{E}S_{l}^{2} \end{array}\right.$$ (5)

In Equation (5), $a^{m}$ and $a^{p}$ denote the inner and outer diameters of the model and prototype structural supports, respectively. $D^{m}$ and $D^{p}$ denote the diameters of the model and prototype structural supports, respectively. The calculation process of the final $D^{m}$ and $a^{m}$ is shown in Equation (6).


$$\left\{ \begin{array}{c} D^{m}=S_{l}D^{p}\\ a^{m}=a^{p} \end{array}\right.$$ (6)

The SecS of slant support for this structure agrees with the calculation. According to Equation (4), the beam and column SecS of the steel section can be further calculated, which is shown in Equation (7).


$$\left\{ \begin{array}{c} \frac{b^{m}{\left(h^{m}\right)}^{3}-\left(b^{m}-t_{w}^{m}\right){\left(h_{w}^{m}\right)}^{3}}{b^{p}{\left(h^{p}\right)}^{3}-\left(b^{p}-t_{w}^{p}\right){\left(h_{w}^{p}\right)}^{3}}=S_{E}S_{l}^{4}\\ \frac{b^{m}h^{m}-\left(b^{m}-t_{w}^{m}\right)\left(h_{w}^{m}\right)}{b^{p}h^{p}-\left(b^{p}-t_{w}^{p}\right)\left(h_{w}^{p}\right)}=S_{E}S_{l}^{2} \end{array}\right.$$ (7)

In Equation (7), $b^{m}$, $h^{m}$, $t_{w}^{m}$, and $h_{w}^{m}$ denote the beam-column flange width, section height, web thickness, and web height of the model, respectively. $b^{p}$, $h^{p}$, $t_{w}^{p}$, and $h_{w}^{p}$ denote the beam-column flange width, section height, web thickness, and web height of the prototype, respectively. Finally, the affected performance parameters of the model are determined by comparing with similar principles [26]. Finally, the strength and stability checking process of the prototype structure is shown in Equation (8).


$$\frac{N^{p}}{A^{p}}\pm \frac{M_{x}^{p}}{\gamma_{x}W_{x}^{p}}\pm \frac{M_{y}^{p}}{\gamma_{y}W_{y}^{p}}\leq f$$ (8)

In Equation (8), $N^{p}$, $M_{x}^{p}$, $M_{y}^{p}$ denote the pressure, bending moment around the X-axis and Y-axis of the prototype, respectively. f denotes the strength design value. $A^{p}$ denotes the pressurized area. $\gamma_{x}$ and $\gamma_{y}$ denote the coefficient of plastic development of the section. $W_{x}^{p}$, $W_{y}^{p}$ denote the resistance moments of the prototype. A total of two different bracing arrangements are designed for the study. One is the continuous X-type center support on one side that is positioned along the model’s X-axis. Secondly, the X-type center support is arranged continuously along the X and Y-axis of the model. The height of the space frame determined by the study is 490 mm and the spacing of the frame columns is 650 mm. Table 1 displays the model’s and the prototype’s steel frame dimensions.

**Table 1.** Steel Frame Dimensions of Prototype and Model.

Member	Steel type		Cross-sectional area (cm^2^)	Second moment of area (cm^4^)
Structural beams	Prototype	HN1000*300*19*36	395.0	62639.0
	Model	HN100*50*5*7	11.9	191.0
Structural column	Prototype	HN1000*300*19*36	395.0	62639.0
	Model	HN100*50*5*7	11.9	191.0
Structural column support components	Prototype	Ф200 × 8	98.5	1.5
	Model	Ф32 × 2.5	4.8	0.1

**Fig 2:**
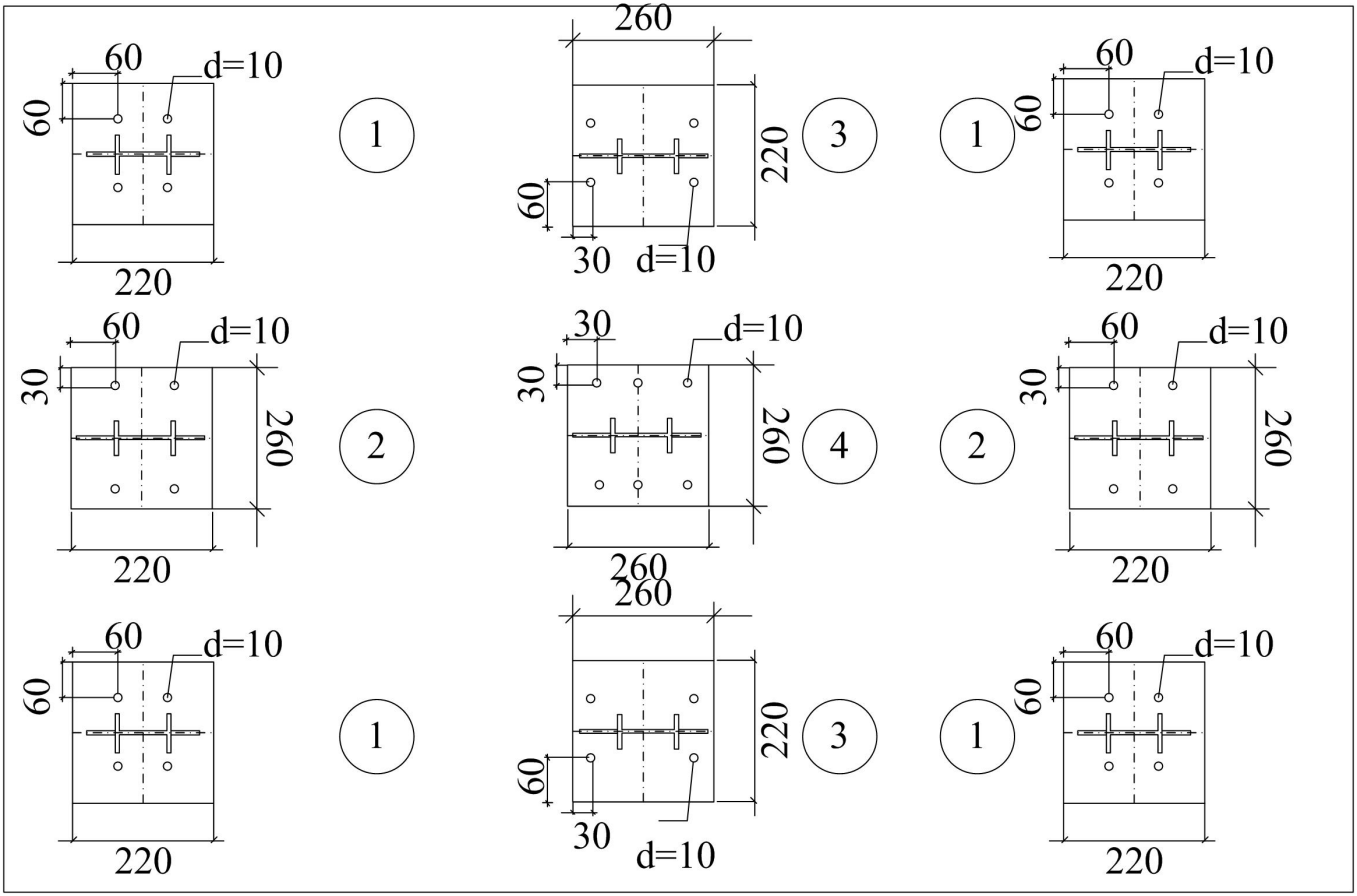
Layout Plan of Structural Column Base Plate.

Referring to the *Code for the Design of Steel Structures (GB50017–2003)*, the connections of beam and column members in the main body of the model designed by the study are all-welded. According to the planar bolt arrangement of the shaking table, the study designs a total of four different structural column footing base plates, and the planar layout forms are shown in Fig 2.

**Fig 3:**
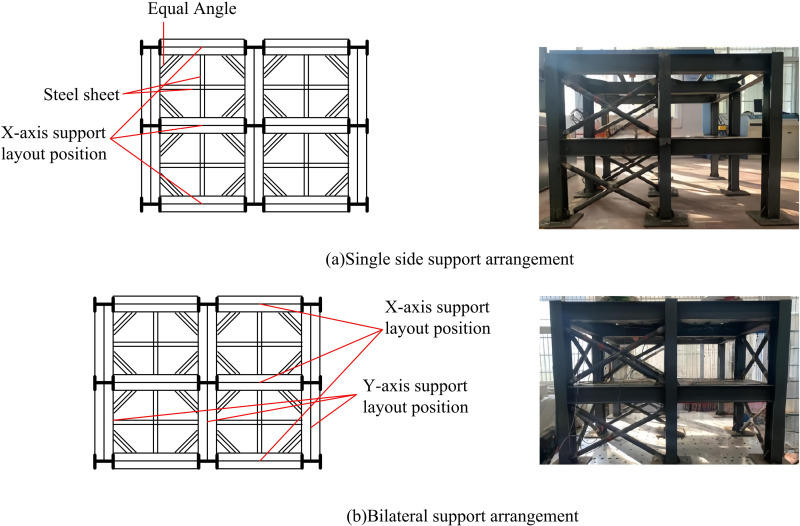
Different Support Layout Forms.

In Fig 2, the SecS of the base plate type I is 220 × 220 × 10 mm, which is distributed in the four corners of the shaker. The SecS of the base plate type II is 220 × 260 × 10mm, distributed on the left and right sides of the shaker. The SecS of the base plate type III is 260 × 220 × 10mm and is distributed on the upper and lower sides of the shaker. The SecS of the base plate type III is 260 × 260 × 10 mm and is distributed in the center of the shaker. The SecS of the 18 stiffening ribs is 70 × 50 × 10mm, and the stiffening ribs and the base plate are both made of Q235 steel. The two forms of bracing arrangement designed by the study are shown in Fig 3.

In Fig 3, the study simplifies the floor slab as equilateral angle steel support, SecS is 40 × 40 × 3mm. Moreover, the structure is equipped with steel sheet for carrying counterweight, SecS is 40 × 6mm, all of which are Q235 steel. The X-type center support welded pipe SecS is Ф32 × 2.5, and the node plate SecS is 50 × 50 × 8mm. Referring to the *Code for the Design of Steel Structures (GB50017–2003)*, it is required that the angle of the node plate of the SSF connected with the support is more than 30°.

### 2.2. Design and numerical simulation analysis of vibration table experiment for scaled model of spatial steel frame

**Fig 4:**
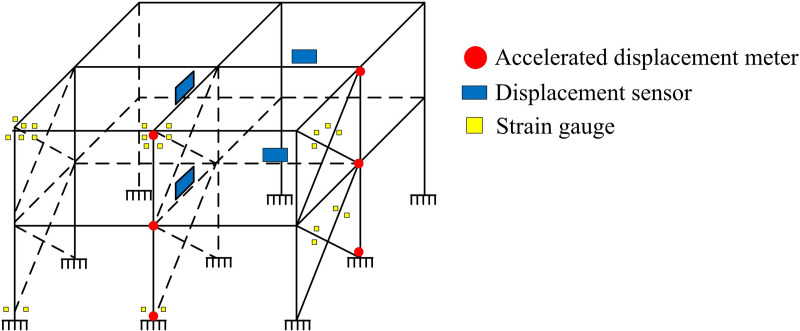
Layout Diagram of Accelerometers, Displacement Sensors, and Resistance Strain Gauges.

After completing the SM design, the study combines VTT and FES to carry out the experimental analysis. A 20-ton electrodynamic shaker is selected for the experiment. The shock excitation force of the electrodynamic shaker is 500kN (pulse width 6ms), the frequency range is 2–2000Hz, the maximum no-load acceleration is 980m/s^2^, and the maximum velocity is 2m/s. The maximum load of the vertical table is 1800 kg, the maximum displacement P-P is 76mm, the permissible tilting moment is 219,000N-m, and the dimensions of the horizontal table are 1500 × 1500mm. In order to measure the seismic response under seismic action, the SSF uses accelerometers, displacement meters, and resistance strain gauges (SG) for measurement and recording. The layout is shown in Fig 4.

In Fig 4, the strain data is collected using resistance SGs. The strain changes are converted to resistance changes and recorded by SGs. A total of 28 SGs are pasted on the beam-column end (BCE) of SSF, which are arranged on the bottom column footing, the upper and lower flanges of the BCE of the 1st floor, the inner and outer flanges of the BCE of the 2nd floor, and the upper and lower supports of the 1st and 2nd floors, respectively. Before pasting the SGs, the SGs are first checked to ensure that they are intact, and then the position of the gauges is cleaned with alcohol to ensure that the surface of the steel frame is free of dust. After applying the glue, the SG is accurately positioned at the patch location and pressed with fingers to squeeze out the excess glue and air bubbles on the surface of the patch and steel [27]. Finally, epoxy resin is applied on the surface of the SGs to prevent damage to the gauges, and the experiments are conducted after waiting for the gauges to dry naturally. The study uses the 1/4 bridge method to connect the SGs to the SGs. The accelerometers are arranged at the center of the web of the nodal columns respectively. The sensitivity of the accelerometers is 500mv/g. The displacement transducers are arranged at the upper end of the midspan beams at different layers.

The selection of seismic waves should first meet the specific requirements of the vibration experiment (VE), including the intensity, direction and frequency characteristics of the simulated earthquake. At the same time, the selection of seismic waves should consider the dynamic characteristics of the steel frame structure to ensure that the selected seismic waves can effectively excite the structure and trigger the expected damage. Considering the SP evaluation of the structure, the effective peak value, spectral characteristics and vibration duration of the SWs are selected for comprehensive consideration. The effective peak value ensures that the seismic simulation effectively excites the structure and triggers damage. To assess how the structure reacts to seismic stimulation, the spectral properties of the SW should coincide with the real earthquake and encompass the major resonance frequency of the structure. Suitable vibration hold-ups can more realistically simulate the seismic process [28,29]. The conversion process of seismic data in the scaled model involves the scaling of time, acceleration, and frequency to scale the time scale, peak acceleration, self-oscillating frequency, and waveform of seismic waves according to the similarity relationship; at the same time, it completes the matching of response spectra, and uses the response spectra to guide the selection and scaling of seismic waves to ensure that the selected seismic waves can produce a response similar to that of the real earthquakes in the simulation.

The study is based on the Pacific Earthquake Engineering Research (PEER) download of seismic information for the prototype structure. The test waveforms are amplified to the target peak value using the SeismoSignal waveform processor. Then the response spectrum curves with a damping value of 5.0% are obtained based on the Chinese code response spectrum generation software. Finally, the loaded SWforms are processed according to the similarity relationship, and the loaded SWform action duration and response acceleration are adjusted [30].

The study uses Ansys software to complete the numerical analysis of the model. In order to corroborate with the VTT, the bracing arrangement of the model during the FES process and the loading conditions are kept consistent with the VTT. Finite element analysis requires accurate material parameters as inputs to provide the necessary basis for structural design and force analysis in order to predict the seismic performance of the modeled structure under seismic action. Input parameters typically include the modulus of elasticity, yield strength, and tensile strength of the steel. Steel material properties tests provide the key parameters needed for modeling and determining strength specifications; they also verify that the material complies with standards and ensure the accuracy of testing and analysis. Therefore, before numerical simulation, the study designs the tensile property experiment of steel with reference to *GB/T228.1-2010 Tensile Test of Metallic Materials* and *GB/T2975-2018 Sampling Position and Specimen Preparation for Mechanical Property Test of Steel and Steel Products*. The experiment uses a universal testing machine to complete the tensile of standard specimens, adopts resistance SGs to record the strain changes, draws the stress-strain curve of steel, and records the relevant material parameters. The accurate setting of material parameters can ensure the accuracy of numerical calculation and the restoration of seismic response. The material property parameters of the steel are shown in Table 2.

**Table 2.** Steel tensile property parameters.

Length	Width	Thickness	Original Scale	Elastic modulus	Maximum force
100mm	5.18mm	15.02mm	50.00mm	1.97 × 10^5^MPa	36.18KN
Tensile strength	Lower yield force	Lower yield strength	Non-proportional elongation strength	/	/
470.00MPa	24983.16N	320.16MPa	325MPa	/	/

The study uses the bifold model as an ontological model for the steel material. Moreover, the material strength, Poisson’s ratio and modulus of elasticity determined from the steel tensile test are inputted into the FE engineering data setup module as material parameters. The calculation of the first fold of the bifold model is shown in Equation (9).


$$\varepsilon_{s}\leq \varepsilon_{y},\sigma_{s}=E_{s}\varepsilon_{s}$$ (9)

In Equation (9), $\varepsilon_{y}$ denotes the strain corresponding to the endpoint of the first fold. $E_{s}$, $\varepsilon_{s}$, and $\sigma_{s}$ denote the modulus of elasticity, strain and stress, respectively. The second segment of the folded line is calculated in Equation (10).


$$\varepsilon_{y}\leq \varepsilon_{s}\leq \varepsilon_{su},\sigma_{s}=f_{y}+\left(\varepsilon_{s}-\varepsilon_{y}\right)\frac{f_{su}-f_{y}}{\varepsilon_{su}-\varepsilon_{y}}$$ (10)

**Fig 5:**
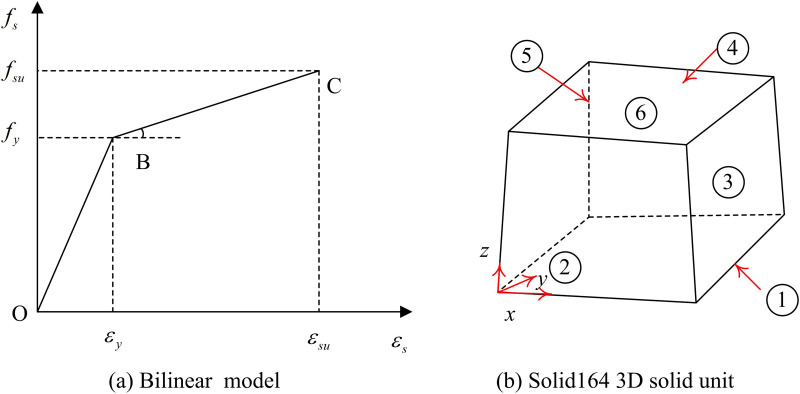
Finite Element Simulation of Constitutive Model and Three-dimensional Solid Elements of Structure.

In Equation (10), $f_{y}$ denotes the material strength. $\varepsilon_{su}$ denotes the strain corresponding to the endpoint of the second segment of the fold line. $f_{su}$ is the strength corresponding to the endpoints of the second section of the fold line. The 3D solid unit of the intrinsic model and structure is shown in Fig 5.

**Fig 6:**
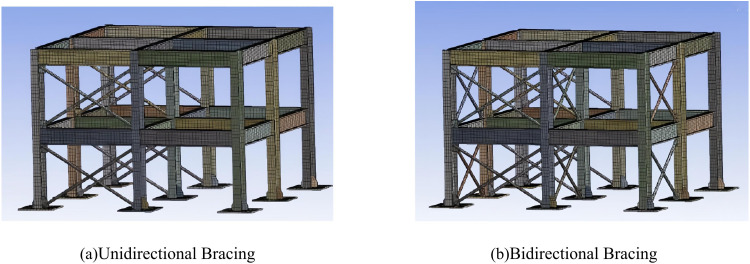
Model diagram of supported steel structure and meshing effect.

As seen in Fig 5, the structural unit in the numerical model is a solid164 3D solid unit. It contains 8 nodes, each representing 9 degrees of freedom. The nodes have translations, accelerations and velocities in the x, y and z directions. The study uses the MultiZone meshing technique in Ansys software to complete the meshing of the model. MultiZone can decompose the irregular solid structure in the steel frame columns into freely delineated mesh regions. The two support models and their meshing effects are shown in Fig 6.

**Fig 7:**
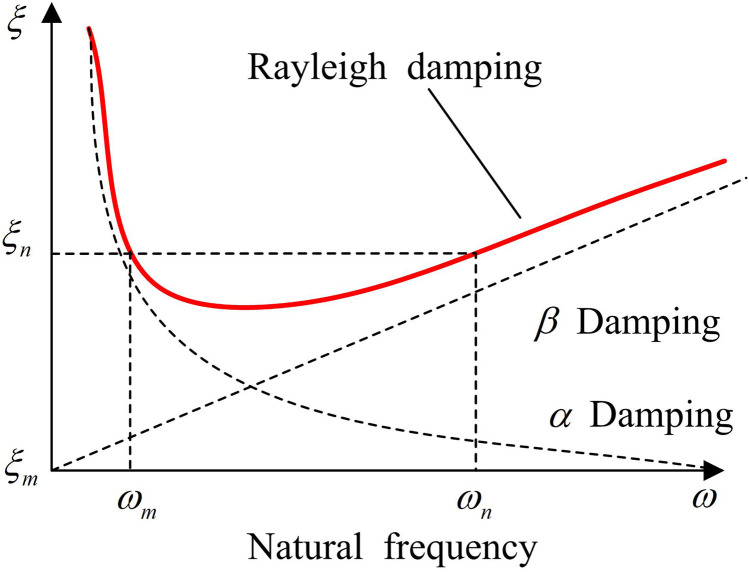
Schematic Diagram of Rayleigh Damping Relationship.

In order to ensure the formation of mutual corroboration between the finite element simulation results and the vibration experiments, and to ensure the consistency between the vibration experiments and the numerical simulation, the study sets the boundary condition constraints on the model according to the VTT. Since the SM is bolted to the shaking table, the study sets the base plate of the numerical model of the steel frame columns as a fixed end, restricting its degrees of freedom in the x, y, and z directions. Meanwhile, the floor slab is set as a rigid region by drawing on common engineering simplification methods, and the mass of the counterweight block is simplified to increase the density of the frame beam. Rayleigh damping is used in the study’s damping analysis to represent the energy dissipation of the numerical model under seismic action. An overview plot of the damping relationship is shown in Fig 7.

The damping matrix [C] for Rayleigh damping is calculated in Equation (11).


[C]=α·[M]+β·[K] (11)

Equation (11) represents the combination of mass matrix and [M] stiffness matrix [K] to obtain the Rayleigh damping matrix. Where α and β denote the damping coefficients of mass and stiffness, respectively. The calculation process is shown in Equation (12).


$$\left\{ \begin{array}{c} \alpha =\frac{2\omega_{1}\omega_{2}\left(\xi_{1}\omega_{2}-\xi_{2}\omega_{1}\right)}{\omega_{2}^{2}-\omega_{1}^{2}}\\ \beta =\frac{2\left(\xi_{2}\omega_{2}-\xi_{1}\omega_{1}\right)}{\omega_{2}^{2}-\omega_{1}^{2}} \end{array}\right.$$ (12)

In Equation (12), $\omega_{1}$ and $\omega_{2}$ denote the circular frequencies of the first two orders of vibration patterns. $\xi_{1}$ and $\xi_{2}$ indicate the values of 0.03 and 0.05, which indicate the damping ratio (DR) for the defense and rare earthquakes.

## 3. Seismic performance analysis of spatial steel frame

The study unfolds the seismic response analysis of space steel frames under seismic action based on VTT with 3D nonlinear FE model.

### 3.1. Vibration table experiment results for spatial steel frame

The study selects El Centro SWs, Taft SWs and Wenchuan SWs in PEER vibration database for experimental comparison with white noise. The study sets up 23 different working situations in total. Table 3 displays the loading of these conditions.

**Table 3.** Specific Loading Conditions under Different Working Conditions.

Working condition number	Seismic wave type	Input acceleration peak	Seismic category	loading direction
1	White noise	50	/	7 degree frequent encounter enhancement
2			/	7 degree defense
3			/	7-degree fortification strengthening
4			/	8 degree defense
5			/	8 degree fortification strengthening
6			/	8 degree rare occurrence
7			/	/
8	El Centro	100*5	X	7 degree defense
9		500	Y	7 degree defense
10		400*5	X	8 degree rare occurrence
11		2000	Y	8 degree rare occurrence
12	Taft	500	X	7 degree defense
13		500	Y	7 degree defense
14		2000	X	8 degree rare occurrence
15		2000	Y	8 degree rare occurrence
16	Wenchuan earthquake wave	250	X	7 degree frequent encounter enhancement
17		500	X	7 degree defense
18		500	Y	7 degree defense
19		750	X	7-degree fortification strengthening
20		1000	X	8 degree defense
21		1500	X	8 degree fortification strengthening
22		2000	X	8 degree rare occurrence
23		2000	Y	8 degree rare occurrence

**Fig 8:**
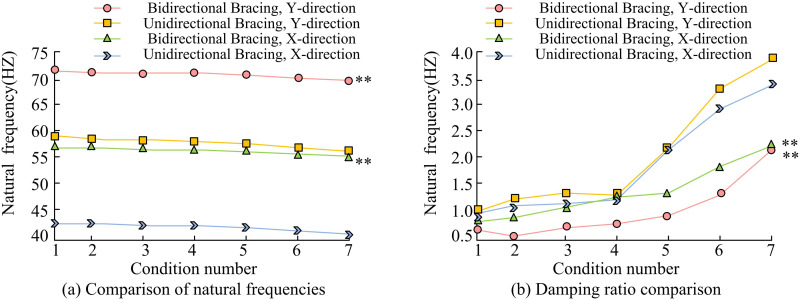
Analysis of Natural Frequency and Damping Ratio. Note: The “*” in the image indicates a significant difference between bidirectional support and unidirectional support at the 0.05 level; “**” indicates that two-way support is significantly different from one-way support at the 0.01 level.

Firstly, the natural frequency (NF) and DR of the model are analyzed on the working conditions 1–7, and the experimental results are shown in Fig 8. The self-oscillation frequency is an important parameter of the dynamic characteristics of the structure, which is related to the stiffness and mass distribution of the structure and reflects the vibration characteristics of the structure under seismic action. Self-oscillation frequency is the frequency of the structure in the free vibration, for good seismic performance of the structure, its self-oscillation frequency is usually low, low frequency vibration on the structure to produce inertia force is small, not easy to cause major damage. This is due to the fact that the structure’s self-oscillation frequency is far away from the dominant frequency of ground shaking, which can avoid the occurrence of resonance phenomenon, thus reducing the amplitude of vibration and the degree of damage to the structure in an earthquake. In Fig 8(a), the initial NF of unidirectional bracing arrangement (UDBA) in X-direction (X-d) and Y-direction (Y-d) are 42.91Hz and 57.98Hz, respectively. The initial NF of bidirectional bracing arrangement (BDBA) in X-d and Y-d are 53.18Hz and 72.49Hz, respectively, indicating that BDBA is more helpful for the overall stiffness of the model. The corresponding excitation accelerations on working conditions 1–7 are 50, 250, 500, 750, 1000, 1500, and 2000gal, respectively. The NFs corresponding to working conditions 1–4 generally show a decreasing trend, but the decreasing magnitude is small, and the maximum decreasing magnitude is only 1.17%. Before the 1500gal excitation acceleration, the model structure shows no obvious damage. After the 1500gal excitation effect, the reduction of NF increases, but the reduction of NF for the structure of BDBA is smaller than that for the structure of UDBA. In conclusion, the self-oscillation frequency of the bidirectionally supported structure was significantly larger (*p* < 0.01) compared to the unidirectionally supported arrangement structure. Damping ratio is a parameter that describes the ability to dissipate energy during structural vibration. Under seismic action, structures need to dissipate the seismic input energy through damping to reduce the magnitude of vibration and damage to the structure. The larger the damping ratio is, the more energy the structure dissipates during vibration and the faster the vibration amplitude decays, thus reducing the response of the structure under seismic action. When the damping ratio is greater than 5%, the vibration of the structure attenuates faster in an earthquake, and the seismic performance is better. In Fig 8(b), the DR of the structure corresponding to working conditions 1–7 under different excitation accelerations shows an increasing trend, indicating that the structure’s own stiffness is significantly affected by the seismic action. However, the DR of the structure in BDBA is smaller than that in UDBA (*p* < 0.01), which shows that the bilaterally braced increases the overall stiffness of the structure. Meanwhile, in the UDBA structure, the DR in the X-d is smaller than that in the Y-d, which shows that the bracing plays the role of SP reinforcement.

**Fig 9:**
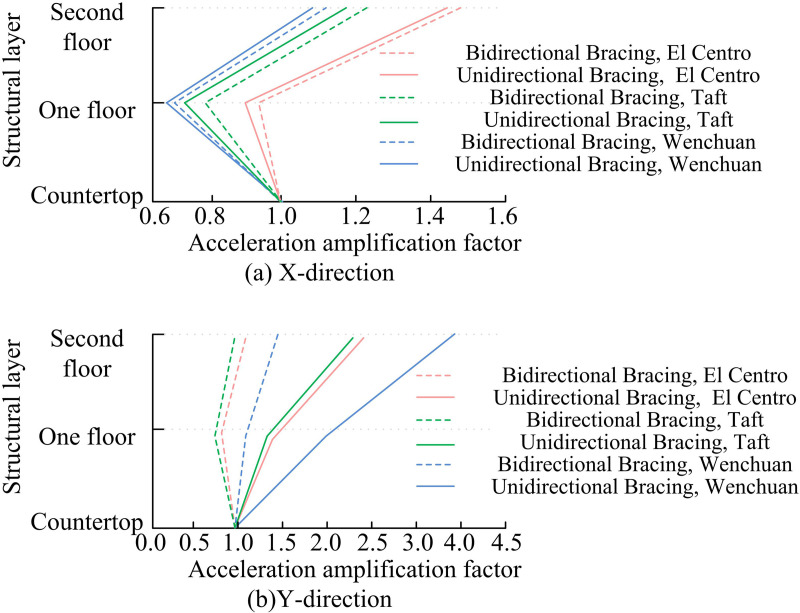
Comparison of Acceleration Amplification Coefficients.

Fig 9 displays the experimental results. The seismic acceleration response of the structure is examined in operating conditions 8–9, 12–13, and 17–18. The acceleration amplification factor is the ratio of the maximum acceleration of the structural response to the maximum acceleration of the bottom input. By analyzing the acceleration amplification factor, it is possible to understand the degree of vibration amplification of a structure during an earthquake and thus assess its seismic performance. The smaller the acceleration amplification factor, the smaller the amplification of ground shaking by the structure, and the smaller the seismic force on the structure. Therefore, structures with smaller acceleration amplification factors usually have better seismic performance. The acceleration amplification factor is generally between 1.0 and 2.5, and when its value is small, the structure has less seismic response and better seismic performance. In Fig 9(a), there is a difference in the degree of influence of different types of SWs on the acceleration amplification factor, and the vibration frequencies of different SWs are different. This is due to the influence of the frequency of seismic waves. Different types of seismic waves have different frequency characteristics, which determine the acceleration amplification effect when they interact with structures or media. At the same time, the amplitude of different seismic waves is not the same, the greater the amplitude of the seismic wave, the greater the energy it carries, and the stronger the destructive force on the structure or medium. In addition, the resonance phenomena will cause the structure’s amplitude to increase when its intrinsic frequency is close to the SW’s frequency, clearly indicating structural degradation. The effect of El Centro on the acceleration amplification factor is the most significant. The acceleration amplification factors of the UDBA structure are 1.000, 0.904, and 1.468 for the table, first floor, and second floor, respectively. In Fig 9(b), there is a significant difference in the Y-d acceleration amplification factor between the unidirectional and bi-directional bracing structures. The BDBA structure produces a smaller acceleration amplification factor, indicating that the bracing arrangement strengthens the SP of the structure.

**Fig 10:**
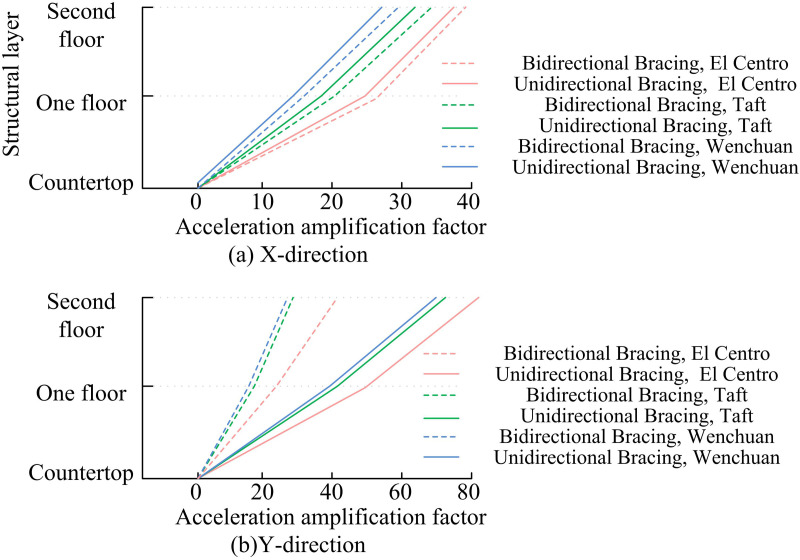
Seismic Displacement Response Analysis.

The structure’s seismic displacement response is examined under operating conditions 10–11, 14–15, and 22–23. Fig 10 displays the outcomes of the experiment. Relative displacement is the amount of displacement of a structure relative to a reference point under seismic action. Relative displacement reflects the deformation of the structure under seismic action and is one of the important indicators for assessing the seismic performance of the structure. The smaller the relative displacement of a structure under seismic action, the smaller its deformation and the more stable the structure. Therefore, structures with smaller relative displacements usually have better seismic performance. The structure’s relative floor displacements exhibit a rising tendency. According to the acceleration amplification factor change regulation, El Centro has the greatest impact on the relative displacement of floors. Moreover, the change of relative displacement in BDBA structure is smaller than UDBA structure in different floors. This indicates that bracing helps to resist the lateral displacement of steel frame structures under seismic action. Furthermore, the relative displacement of the floors of the structures with different bracing arrangements in the Y-d varies more, the relative displacement of the floors in the X-d varies less, and the value of the relative displacement of the BDBA structure in the X-d is larger. This is due to the fact that the BDBA bracing arrangement in the Y-d does not enhance the lateral resilience of the structure in the X-d. Instead, it increases the lateral stiffness of the structure, which in turn impairs the capacity to dissipate energy, resulting in a reduction in the SP and an increase in the relative displacement.

**Fig 11:**
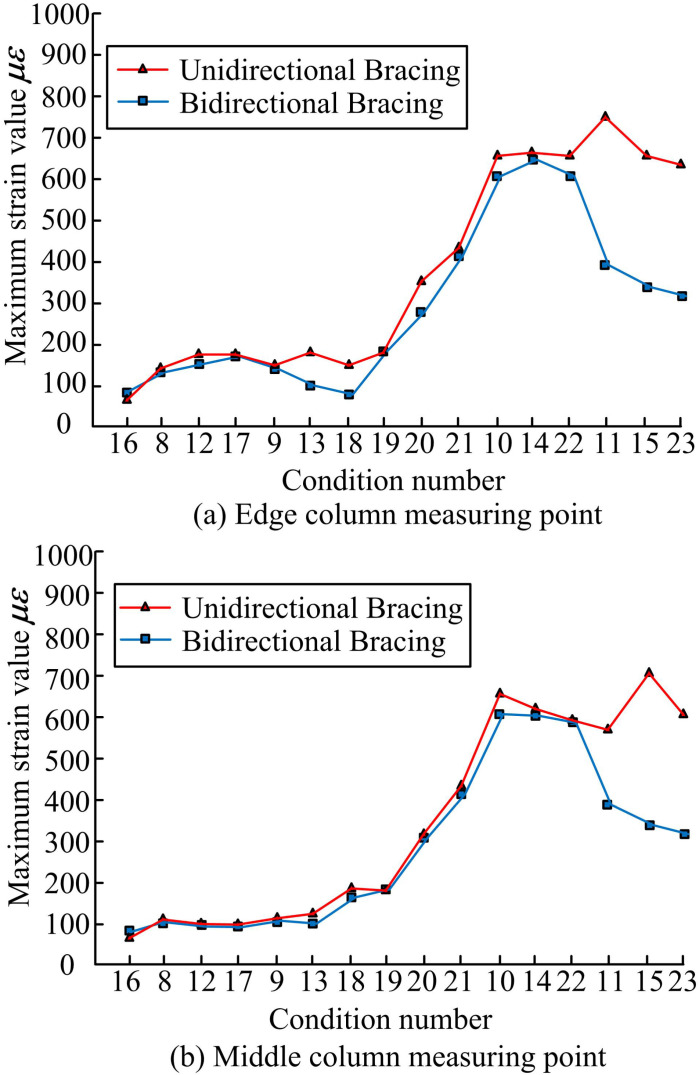
Analysis of Strain Variation Results of Column Base Strain Measurement Points.

The strain change rule of SSF structure under different working conditions is analyzed with the magnitude of SI as the horizontal coordinate. The strain change results of the column foot strain measurement points are analyzed as an example. The strain change is shown in Fig 11. Strain is a physical quantity that describes the change in shape or size of a material or member under stress. Strain reflects the stress on structural materials under seismic action, and is an important indicator for assessing whether a structural member has reached a yield or damage state. Under seismic action, the smaller the strain of a member is, the more stable its stress state is, the less likely it is to be damaged, and it will not easily reach the yielding or damage state, thus ensuring the bearing capacity and seismic performance of the structure. The strain change rule of side columns and center columns under different seismic intensities is consistent. Moreover, the maximum strain value shows an upward trend with the increase of SI. When the loading condition is in X-d, the strain value of the structure does not fluctuate significantly in conditions 8, 12, 17 and 10, 14, 22. When the loading condition is in Y-d, the strain value of the structure changes significantly in conditions 9, 13, 18 and 11, 15, 23. The difference between the maximum strain values of the structures with two different bracing arrangements shows a significant gap. It can be observed that the bidirectional bracing structure with wave following arrangement plays the role of SP, and the maximum strain values show a steep decrease. The difference in the maximum strain values of the two different bracing arrangements is more obvious in the rarefied condition of 8 degrees.

**Fig 12:**
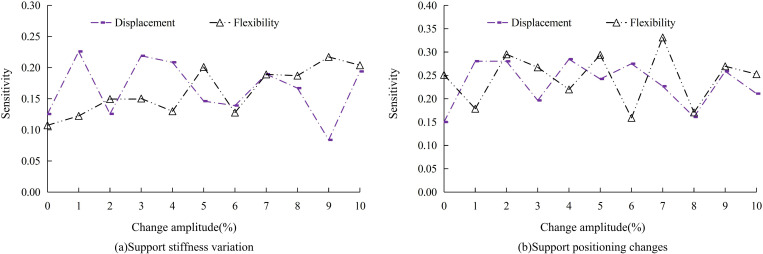
Sensitivity Analysis under Changes in Supporting Structure.

Taking the unidirectional braced structure as an example, the results of the sensitivity analysis of the changes in the stiffness and positioning of the braced structure to the displacements and strains under the condition of rare earthquakes of degree 8 are shown in Fig 12. As seen in Fig 12(a), when the stiffness of the braced structure is changed, the sensitivity change of displacement and strain of the structure fluctuates in the interval of 0.08–0.25; and as seen in Fig 12(b), when the positioning of the braced structure is changed, the interval of sensitivity change increases and fluctuates in the interval of 0.15–0.35. Thus, in contrast, changing the positioning of the bracing will cause greater changes in the seismic performance of the structure.

### 3.2. Comparison of vibration table experiment and finite element numerical modes for spatial steel frame

**Fig 13:**
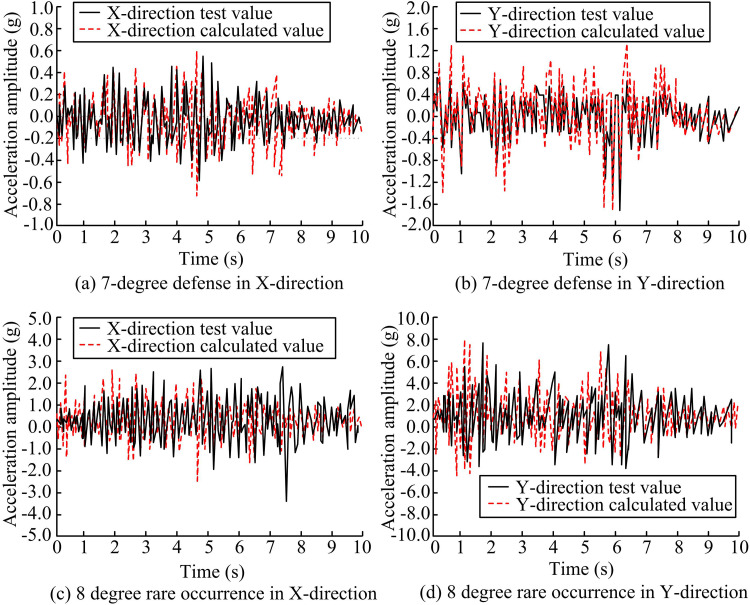
Comparison of Finite Element and Vibration Table Acceleration Time History Curves.

The acceleration time-course curves of the two experimental methods are firstly carried out. The Wenchuan SW is used, and the second-story midspan response values of the UDBA structure under 7-degree fortification and 8-degree rare earthquakes are selected for analysis, respectively. The comparison results are shown in Fig 13. The acceleration time curve is a curve that describes the change of acceleration with time for a structure under seismic action. By analyzing the characteristics of the curves such as shape, amplitude and duration, it is possible to understand the vibration characteristics and stability of the structure during an earthquake. For a structure with good seismic performance, the peak of the acceleration time curve should be smaller and the fluctuation should be smoother. This indicates that the structure is less shocked by the earthquake and is able to absorb and dissipate the seismic energy better. The acceleration curves of FES and VTT under different intensity earthquakes are in good agreement. In Fig 13 (a) and (c), the error trend of X-d acceleration amplitude fitting curves under the action of 8-degree rare earthquakes is larger. However, the curve trend and the corresponding time point of the peak value of the FES structure are generally consistent with the VE results. Taken together, the validity and rationality of the FES experiments are verified.

**Fig 14:**
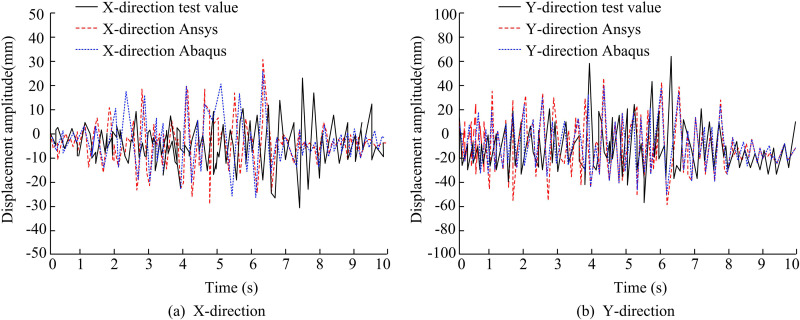
Comparison of Position Time History Curves between Finite Element and Vibration Experiments.

In addition, the study added an assisted validation in the Abaqus simulation software, the displacement time course curves of FE and VE are compared by choosing BDBA structure and 8 degree rare earthquakes. The experimental results are shown in Fig 14. The position time-range curves of FES fit well with the displacement time-range curves obtained from VE. The seismic response data in FE can effectively correspond to the experimental results, indicating that the FE modeling results can effectively reflect the seismic response of the SSF under actual seismic effects. Meanwhile, the simulation results of Abaqus are more similar to the simulation results of the study using Ansys software, which again confirms the reliability of the simulation results.

The results of the comparison between the maximum acceleration and maximum displacement of the FES and the scaled-down experiments are shown in Table 4. The difference between the maximum values of acceleration in X-d and Y-d of FES and VE under 7-degree seismic protection is about 9.0% and 15.0%. The difference between the maximum values of acceleration in X-d and Y-d of FES and VE under 8-degree rare earthquakes is about 7.0% and 19.0%. The results of the maximum acceleration calculated by FE are, on average, smaller than those of VE. This is due to the fact that during the numerical simulation process, the study sets the base plate of the steel frame column as a fixed end, limiting its degrees of freedom in the x, y, and z directions. Under the 7-degree seismic protection, the difference between the maximum displacements of FES and VE in the x- and y-directions is approximately at the level of 4.0% and 7.0%. The difference in the maximum displacements of FES and VE in the X-d and Y-ds under an 8-degree seismic event is approximately 23.0% and 20.0%, respectively. During the maximum displacement analysis, the FE calculation results are overall greater than the VE results, which is due to the boundary conditions of the numerical model being fully cemented. Comprehensively, it can be observed that the FES error exists partially greater than 20%, which is due to the material setting of the numerical simulation, the simplification of the physical model, and the numericalization process of the computational model. However, overall, the simulation results of FE are scientifically sound. In addition, comparing the storey drift ratios of different structures, it can be seen that the storey drift ratio of the structure with selective bi-directional bracing arrangement is smaller, which represents a smaller degree of inter-storey deformation of the structure under horizontal load, and its seismic performance is better.

**Table 4.** Comparison Analysis of Maximum Displacement.

Seismic category	Number of layers	Indicators	Direction	Bracing arrangement form			
				Unidirectional Bracing		Bidirectional Bracing	
				Vibration table	Finite element	Vibration table	Finite element
7 degree defense	One floor	Maximum acceleration value (mm)	X	0.326	0.295	0.352	0.318
			Y	1.084	0.919	0.513	0.435
		Maximum displacement (g)	X	5.556	5.765	5.984	6.214
			Y	12.564	13.464	6.164	6.606
	Second floor	Maximum acceleration value(mm)	X	0.536	0.485	0.556	0.503
			Y	1.964	1.668	0.746	0.634
		Maximum displacement (g)	X	8.943	9.295	9.221	9.575
			Y	22.564	24.170	9.154	9.819
8 degree rare occurrence	One floor	Maximum acceleration value (mm)	X	1.289	1.197	1.346	1.250
			Y	4.682	3.771	2.349	1.888
		Maximum displacement (g)	X	18.264	22.494	19.031	23.436
			Y	38.154	45.846	19.497	23.429
	Second floor	Maximum acceleration value (mm)	X	2.049	1.904	2.164	2.007
			Y	8.264	6.674	3.497	2.792
		Maximum displacement (g)	X	30.164	37.228	31.064	38.410
			Y	69.464	83.801	27.164	32.724
/	/	Interlayer Displacement Ratio	/	1/536	1/598	1/861	1/987

## 4. Conclusion

To gain a deeper understanding of the SP of the SFB system of the SS under seismic action and to improve the overall stiffness and stability of the SS, the study conducted modeling experiments and modeling analysis of the SP of the SFB under seismic action. The experimental results indicated that BDBA contributes to the overall stiffness and lateral movement resistance of the model. Under the same SI, the initial NF of BDBA in X-d and Y-d was improved by 10.27 Hz and 14.51 Hz compared with unidirectional bracing, respectively. The DR, acceleration amplification factor and relative displacement of floors of the BDBA structure were smaller than that of the UDBA. The effect of the El Centro on the acceleration amplification factor and relative displacement was most significant. Overall, the BDBA has a higher structural energy dissipation capacity. The column strain of bidirectional bracing structure will occur a steep decrease of strain value. The validity and reasonableness of the FES experiment was verified by the comparison results of the acceleration time-course curve and displacement time-course curve fitting. Moreover, due to the boundary condition setting of FE, the result of maximum acceleration calculated by FE was smaller than the result of VE, and the result of maximum displacement calculated by FE was larger than the result of VE. This study contributes to the understanding of the SP of the SFB system, but the SP under different SSF parameters under seismic action remains to be studied.

## Supporting information

S1 Data Minimal Data Set Definition. (DOC)
