# Supplementary material for: Seismic performance analysis of braced steel structures based on vibration experiments and finite element simulation
Source: PLoS One. 2025 May 9;20(5):e0322379. doi: 10.1371/journal.pone.0322379 (PMC12064046; doi:10.1371/journal.pone.0322379)
Supplement: S1 Data — (DOC) [file pone.0322379.s001.doc]

**Figure 8 Analysis of Natural Frequency and Damping Ratio (a)**

| Condition number | X | | Y | |
| --- | --- | --- | --- | --- |
|  | Unidirectional Bracing | Bidirectional Bracing | Unidirectional Bracing | Bidirectional Bracing |
| 1 | 42.862 | 55.917 | 58.733 | 71.247 |
| 2 | 42.650 | 55.627 | 58.227 | 71.007 |
| 3 | 42.303 | 55.337 | 57.831 | 70.953 |
| 4 | 42.160 | 55.009 | 57.480 | 70.758 |
| 5 | 41.678 | 54.752 | 56.972 | 70.622 |
| 6 | 40.935 | 54.762 | 56.068 | 69.382 |
| 7 | 40.159 | 53.435 | 55.146 | 68.916 |

**Figure 8 Analysis of Natural Frequency and Damping Ratio (b)**

| Condition number | X | | Y | |
| --- | --- | --- | --- | --- |
|  | Unidirectional Bracing | Bidirectional Bracing | Unidirectional Bracing | Bidirectional Bracing |
| 1 | 0.947 | 0.734 | 0.986 | 0.573 |
| 2 | 1.057 | 0.750 | 1.231 | 0.499 |
| 3 | 1.086 | 1.031 | 1.339 | 0.619 |
| 4 | 1.192 | 1.208 | 1.247 | 0.674 |
| 5 | 2.076 | 1.383 | 2.165 | 0.978 |
| 6 | 2.982 | 1.790 | 3.459 | 1.752 |
| 7 | 3.474 | 2.367 | 3.848 | 2.335 |

**Figure 9 Comparison of Acceleration Amplification Coefficients (a)**

| Placement | Acceleration correlation coefficient | El Centro | | Taft | | Wenchuan | |
| --- | --- | --- | --- | --- | --- | --- | --- |
|  |  | Unidirectional Bracing | Bidirectional Bracing | Unidirectional Bracing | Bidirectional Bracing | Unidirectional Bracing | Bidirectional Bracing |
| Countertops | Peak tabletop input acceleration | 0.505 | 0.509 | 0.486 | 0.480 | 0.499 | 0.498 |
|  | Acceleration amplification factor | 1.000 | 1.000 | 1.000 | 1.000 | 1.000 | 1.000 |
| First layer | Structural response acceleration peak | 0.454 | 0.478 | 0.352 | 0.374 | 0.338 | 0.352 |
|  | Acceleration amplification factor | 0.899 | 0.924 | 0.728 | 0.773 | 0.677 | 0.702 |
| Second layer | Structural response acceleration peak | 0.728 | 0.744 | 0.570 | 0.583 | 0.540 | 0.557 |
|  | Acceleration amplification factor | 1.442 | 1.479 | 1.170 | 1.223 | 1.086 | 1.116 |

**Figure 9 Comparison of Acceleration Amplification Coefficients (b)**

| Placement | Acceleration correlation coefficient | El Centro | | Taft | | Wenchuan | |
| --- | --- | --- | --- | --- | --- | --- | --- |
|  |  | Unidirectional Bracing | Bidirectional Bracing | Unidirectional Bracing | Bidirectional Bracing | Unidirectional Bracing | Bidirectional Bracing |
| Countertops | Peak tabletop input acceleration | 2.005 | 0.979 | 2.016 | 2.024 | 1.999 | 1.998 |
|  | Acceleration amplification factor | 1.000 | 1.000 | 1.000 | 1.000 | 1.000 | 1.000 |
| First layer | Structural response acceleration peak | 4.254 | 2.188 | 4.258 | 2.231 | 4.661 | 2..369 |
|  | Acceleration amplification factor | 2.109 | 1.124 | 2.134 | 1.094 | 2.316 | 1.169 |
| Second layer | Structural response acceleration peak | 7.628 | 3.054 | 7.708 | 3.026 | 3.211 | 3.131 |
|  | Acceleration amplification factor | 3.742 | 1.539 | 3.861 | 1.561 | 4.261 | 1.649 |

**Figure 10 Seismic Displacement Response Analysis**

| Placement | Directional | El Centro | | Taft | | Wenchuan | |
| --- | --- | --- | --- | --- | --- | --- | --- |
|  |  | Unidirectional Bracing | Bidirectional Bracing | Unidirectional Bracing | Bidirectional Bracing | Unidirectional Bracing | Bidirectional Bracing |
| Countertops | X | 0.000 | 0.000 | 0.000 | 0.000 | 0.000 | 0.000 |
|  | Y | 0.000 | 0.000 | 0.000 | 0.000 | 0.000 | 0.000 |
| First layer | X | 23.436 | 24.840 | 19.572 | 20.258 | 18.101 | 19.032 |
|  | Y | 45.942 | 23.709 | 39.860 | 20.284 | 38.616 | 19.931 |
| Second layer | X | 38.712 | 39.730 | 31.204 | 32.805 | 30.381 | 30.469 |
|  | Y | 80.796 | 32.170 | 71.589 | 28.982 | 69.801 | 27.360 |

**Figure 11 Analysis of Strain Variation Results of Column Base Strain Measurement Points**

| Condition number | Edge column measuring point | | Middle column measuring point | |
| --- | --- | --- | --- | --- |
|  | Unidirectional Bracing | Bidirectional Bracing | Unidirectional Bracing | Bidirectional Bracing |
| 16 | 87.043 | 93.242 | 87.542 | 94.546 |
| 8 | 122.677 | 137.647 | 104.200 | 103.953 |
| 12 | 189.185 | 171.067 | 103.927 | 101.656 |
| 17 | 189.346 | 181.993 | 105.193 | 102.890 |
| 9 | 159.560 | 158.693 | 108.900 | 104.567 |
| 13 | 184.002 | 108.258 | 119.999 | 103.919 |
| 18 | 153.079 | 98.325 | 194.206 | 178.422 |
| 19 | 197.820 | 195.554 | 191.371 | 191.680 |
| 20 | 341.982 | 295.765 | 304.153 | 305.694 |
| 21 | 416.765 | 412.996 | 418.757 | 403.285 |
| 10 | 663.018 | 605.557 | 649.328 | 608.219 |
| 14 | 679.739 | 667.539 | 618.755 | 600.862 |
| 22 | 669.705 | 600.365 | 604.703 | 602.903 |
| 11 | 746.689 | 409.175 | 583.226 | 398.349 |
| 15 | 650.455 | 323.878 | 706.022 | 329.216 |
| 23 | 612.403 | 310.271 | 606.358 | 309.671 |

**Figure 12 Sensitivity Analysis under Changes in Supporting Structure**

| Support stiffness variation | | | Support positioning changes | | |
| --- | --- | --- | --- | --- | --- |
| Change amplitude(%) | Displacement | Flexibility | Change amplitude(%) | Displacement | Flexibility |
| 0 | 0.125 | 0.107 | 0 | 0.150 | 0.251 |
| 1 | 0.226 | 0.122 | 1 | 0.280 | 0.179 |
| 2 | 0.126 | 0.150 | 2 | 0.280 | 0.295 |
| 3 | 0.219 | 0.150 | 3 | 0.196 | 0.267 |
| 4 | 0.209 | 0.130 | 4 | 0.285 | 0.220 |
| 5 | 0.146 | 0.201 | 5 | 0.242 | 0.294 |
| 6 | 0.139 | 0.128 | 6 | 0.275 | 0.159 |
| 7 | 0.189 | 0.189 | 7 | 0.227 | 0.331 |
| 8 | 0.167 | 0.187 | 8 | 0.161 | 0.171 |
| 9 | 0.084 | 0.217 | 9 | 0.259 | 0.270 |
| 10 | 0.193 | 0.204 | 10 | 0.211 | 0.253 |

**Figure 13 Comparison of Finite Element and Vibration Table Acceleration Time History Curves**

| Time (s) | Acceleration amplitude (g)  7-degree defense | | | | Acceleration amplitude (g)  8 degree rare occurrence | | | |
| --- | --- | --- | --- | --- | --- | --- | --- | --- |
|  | Test value | | Calculated value | | Test value | | Calculated value | |
|  | X | Y | X | Y | X | Y | X | Y |
| 0 | 0.056 | 0.232 | -0.067 | 0.243 | 0.782 | 1.234 | 0.893 | 1.139 |
| 1 | 0.178 | -0.627 | 0.179 | -0.649 | 0.349 | 3.610 | 0.241 | 3.037 |
| 2 | 0.151 | -0.436 | 0.163 | -0.416 | 0.615 | 5.292 | 0.713 | 5.628 |
| 3 | 0.186 | 0.048 | -1.292 | 0.065 | 0.512 | 4.976 | 0.800 | 5.856 |
| 4 | -0.103 | 0.748 | 0.210 | 1.320 | 0.587 | 2.943 | 0.431 | 2.663 |
| 5 | -0.199 | 1.246 | 0.304 | 0.210 | 0.172 | 2.041 | 0.266 | 2.802 |
| 6 | 0.173 | 0.458 | 0.183 | 0.414 | 0.634 | 5.790 | 0.509 | 5.203 |
| 7 | -0.149 | 0.313 | -0.187 | 0.388 | 0.905 | 5.356 | 0.867 | 5.984 |
| 8 | 0.167 | 0.184 | 0.145 | 0.314 | 0.416 | 5.178 | 0.322 | 5.374 |
| 9 | -0.157 | 0.352 | -0.166 | 0.314 | 0.222 | 2.677 | 0.278 | 2.390 |
| 10 | -0.070 | 0.289 | -0.106 | 0.265 | 0.515 | 3.706 | 0.554 | 3.009 |

**Figure 14 Comparison of Position Time History Curves between Finite Element and Vibration Experiments**

| Time (s) | Acceleration amplitude (g)  8 degree rare occurrence | | | | | |
| --- | --- | --- | --- | --- | --- | --- |
|  | Test value | | Ansys | | Abaqus | |
|  | X | Y | X | Y | X | Y |
| 0 | -5.056 | -16.612 | -7.591 | -11.510 | -6.703 | -10.154 |
| 1 | 2.351 | -20.326 | 2.513 | -24.717 | 2.213 | -22.366 |
| 2 | 9.040 | -3.741 | 8.835 | -4.791 | 7.585 | -3.117 |
| 3 | 11.790 | -15.243 | 12.184 | -15.468 | 13.067 | -15.995 |
| 4 | -5.294 | 21.546 | -20.983 | 23.535 | -22.523 | 23.391 |
| 5 | -6.667 | 17.461 | -11.294 | 15.029 | -11.372 | 16.836 |
| 6 | -15.990 | 46.293 | -16.533 | -9.880 | -16.166 | -8.987 |
| 7 | -5.988 | 16.293 | -2.499 | -19.254 | -2.793 | -16.749 |
| 8 | -2.647 | -30.942 | -6.082 | -30.244 | -7.613 | -30.099 |
| 9 | -4.771 | -30.339 | -2.976 | -11.833 | -3.492 | -11.134 |
| 10 | -2.064 | -31.007 | -0.655 | -15.828 | -0.673 | -16.771 |
